# Delays in the decision to seek care and associated factors among mothers who delivered in rural health centers in Wolaita Zone, Southern Ethiopia

**DOI:** 10.3389/fgwh.2023.1236242

**Published:** 2024-01-11

**Authors:** Kelemu Abebe Gelaw, Yibeltal Assefa Atalay, Firehiwot Zerefu, Natnael Atnafu Gebeyehu

**Affiliations:** ^1^ School of Midwifery, College of Health Science and Medicine, Wolaita Sodo University, Wolaita Sodo, Ethiopia; ^2^ School of Public Health, College of Health Science and Medicine, Wolaita Sodo University, Wolaita Sodo, Ethiopia

**Keywords:** first maternal delay, associated factors, rural health center, Wolaita Sodo, Ethiopia

## Abstract

**Background:**

Delays in seeking care in health facilities during pregnancy and childbirth can potentially lead to adverse outcomes for women with obstetric complications. These complications lead to maternal mortality and morbidity in developing countries such as Ethiopia. The magnitude and underlying causes of maternal first delay in Ethiopia, particularly in rural areas of the country, are not well documented. This study aims to assess the magnitude of delay in the decision to seek care and associated factors among mothers who gave birth in rural health centers in Wolaita Zone, Southern Ethiopia.

**Method and materials:**

A facility-based cross-sectional study was undertaken among mothers who gave birth in rural health centers of Wolaita Zone, Southern Ethiopia, from 30 June 2020 to 30 July 2022. A sample size of 410 study participants was selected from each public health center using the systematic random sampling method. Data were collected from a pretested and structured questionnaire using an Open Data Kit; analysis was carried out using SPSS version 25. The determining factors for the first delay were identified using binary logistic regression. Variables with a *p*-value of less than 0.25 in binary analysis were selected for a multivariable analysis. Variables with a *p*-value of less than 0.05 were considered statistically significant.

**Results:**

The magnitude of delay in the decision to seek care in health facilities was 42.1% among mothers who gave birth in rural health centers in the Wolaita Zone, Southern Ethiopia. Unemployed mothers (Adjusted Odd Ratio, 2,529; 95% CI, 1.546, 4.136), husbands with no formal education (Adjusted Odd Ratio, 1.290; 95% CI, 1.029, 1.616), mothers who had negative attitudes towards seeking care in health facilities, and (Adjusted Odd Ratio; 1.695; 95% CI, 1.061, 2.709) were significantly associated with a delay in the decision to seek care at a health facility.

**Conclusion:**

The magnitude of the first maternal decision to seek care at health facilities among mothers was high in the study area. Efforts should be made to strengthen the literacy level of the husbands of mothers and increase household income through various income-generating approaches. In addition, the dissemination of health information could effectively raise community awareness of the importance of institutional delivery.

## Introduction

Every day, an estimated 810 mothers die worldwide because of complications related to pregnancy or childbirth. Sub-Saharan Africa is severely affected by maternal deaths and accounts for 86% of all maternal deaths in the world. Most of these deaths occur during childbirth or immediately postpartum, highlighting the urgent need for improved maternal healthcare in this region (1, 2).

Thaddeus and Maine's three-lag model, formulated in 1994, facilitates the identification of indirect determinants that contribute to maternal mortality. This model highlights three crucial phases: the first delay, where the decision to seek care at a health facility is delayed (one of the factors that contribute to the high maternal mortality rate in developing countries); the second delay, which relates to identifying and reaching the health facility; and the third delay, which involves the delay in care at the health facility (3). Delay in the decision to seek delivery in health facilities during pregnancy and birth can potentially lead to adverse outcomes for women with obstetric complications, severe bleeding, premature rupture of membranes, obstructed labor, and obstetric fistulas. These complications, which commonly occur during pregnancy and childbirth, are known to contribute significantly to maternal mortality (4). Delay in the decision to seek delivery at health facilities is related to the time needed to decide on emergency care, which occurs within the family and community level (5).

Women living in rural areas face significant challenges in accessing adequate healthcare because of the lack of qualified health professionals in these areas (6). Only 46% of women living in low-income countries receive professional obstetric care, despite the advances made in antenatal care in various regions of the world over the last decade (7). This prevalence shows that millions of births take place without the presence of a midwife, doctor, or trained nurse. Poverty, geographic isolation, limited access to information, inadequate healthcare, and cultural norms represent barriers that prevent women from receiving appropriate medical care during pregnancy and childbirth (8).

Maternal deaths can be averted through early detection and intervention. However, several factors can delay women's access to emergency obstetric care. In sub-Saharan Africa, a variety of interrelated barriers have been identified that contribute to women's delay in seeking emergency obstetric care. These barriers manifest at different levels and include the home, transportation to healthcare facilities, and the healthcare facilities themselves (9, 10).

There are a variety of factors that contribute to maternal health complications resulting from the lack of proactive decision-making in seeking medical care in health facilities. These factors are closely related to inadequate provision of antenatal care, age differences, educational differences, and employment status (11). In addition, studies have reported that factors at both the individual and the household levels lead to difficulties in seeking appropriate obstetric care. These factors include the lack of transportation or difficulty in arranging transportation, financial constraints, younger age, illiteracy, lower income, unemployment, limited utilization of health services, unplanned pregnancy, husband's educational status, inadequate knowledge of obstetric danger signs, and cultural beliefs (12, 13). Differences in first maternal delay in the decision to seek care at health facilities in Ethiopia revealed differences among different sites. In particular, Hadiya Zone public health facilities recorded a rate of magnitude of 40.1% (14), while in Bahir Dar city, it was 37.8% (15). Furthermore, a rate of magnitude of maternal delays of 46.8% was observed in public health facilities of the Gamo Zone (16).

The United Nations Sustainable Development Goals and the Global Strategy for the Health of Women, Children, and Adolescents (2016–2030) emphasize achieving optimal health and wellbeing, including for mothers and newborns, such as creating supportive environments, to prevent avoidable maternal and perinatal deaths (17, 18). In 1990, the World Health Organization (WHO) released data showing that the maternal mortality rate in Ethiopia was 1,250 deaths per 100,000 live births. However, there has been a significant development since then, as this rate has fallen by a remarkable 70%. According to recent reports, there are only 353 maternal deaths per 100,000 live births in Ethiopia (19, 20).

Efforts have been made to improve health extension programs nationwide to increase the overall coverage of maternal health services in Ethiopia. However, there is still a lack of general access to maternal health services, particularly skilled birth attendants. The Mini-Ethiopian Demographic Health System (Mini-EDHS) 2019 shows that home deliveries remain prevalent in Ethiopia, particularly in rural areas (21). Therefore, it is important to find factors that cause delays in the decision to seek delivery at health facilities for reducing maternal mortality related to home birth in Ethiopia, especially in rural areas. Hence, this study aims to assess the magnitude of delay in the decision to seek care in health facilities and associated factors among mothers who gave birth at rural health centers in Wolaita Zone, Southern Ethiopia.

## Method and materials

### Study setting and period

This study was conducted between 15 June and 30 July 2022, in selected districts within the Wolaita Zone. The zone's capital, the city of Sodo, is located 330 km south of Addis Ababa and 165 km from Hawassa. The Wolaita Zone covers 12 rural woredas and six municipalities with a total population of 5,385,782, of which 2,746,749 are women. The reproductive age group is estimated at 639,992 (23.3%), and the expected annual delivery in health facilities in the zone is 71,524. The zone has one general hospital, 21 district hospitals, seven primary hospitals, and 72 health centers, 50 of which are in rural areas.

### Study design

A facility-based multicenter cross-sectional study design was employed.

### Population

The study included all mothers who delivered in rural health centers in the Wolaita Zone as its source population. The study population, on the other hand, consisted of randomly selected mothers who delivered in a rural health center within 24 h in the same zone during the data collection period.

### Eligibility criteria

Mothers who had given birth at a rural health center during the data collection period in the health center were included in the study. However, mothers who had been referred for further evaluation and care and mothers who had been admitted before the onset of labor due to unrelated medical factors were excluded.

### Sample size determination

The sample size for this study was determined with Open Epi software version 3.0.1 using the single population proportion calculation method. The assumptions made were a confidence interval of 95%, a margin of error of 0.05, and a specific expected Prevalence (P), based on a study carried out in the nearby Gamo Zone, where the rate of the magnitude of the first maternal delay was established at 46.8%. Consequently, a sample size of 381 was determined. To account for a potential non-response rate of 10%, the sample size was increased to 410 women, which ultimately represented the final sample size for the study.

### Sampling procedures

**Figure 1:**
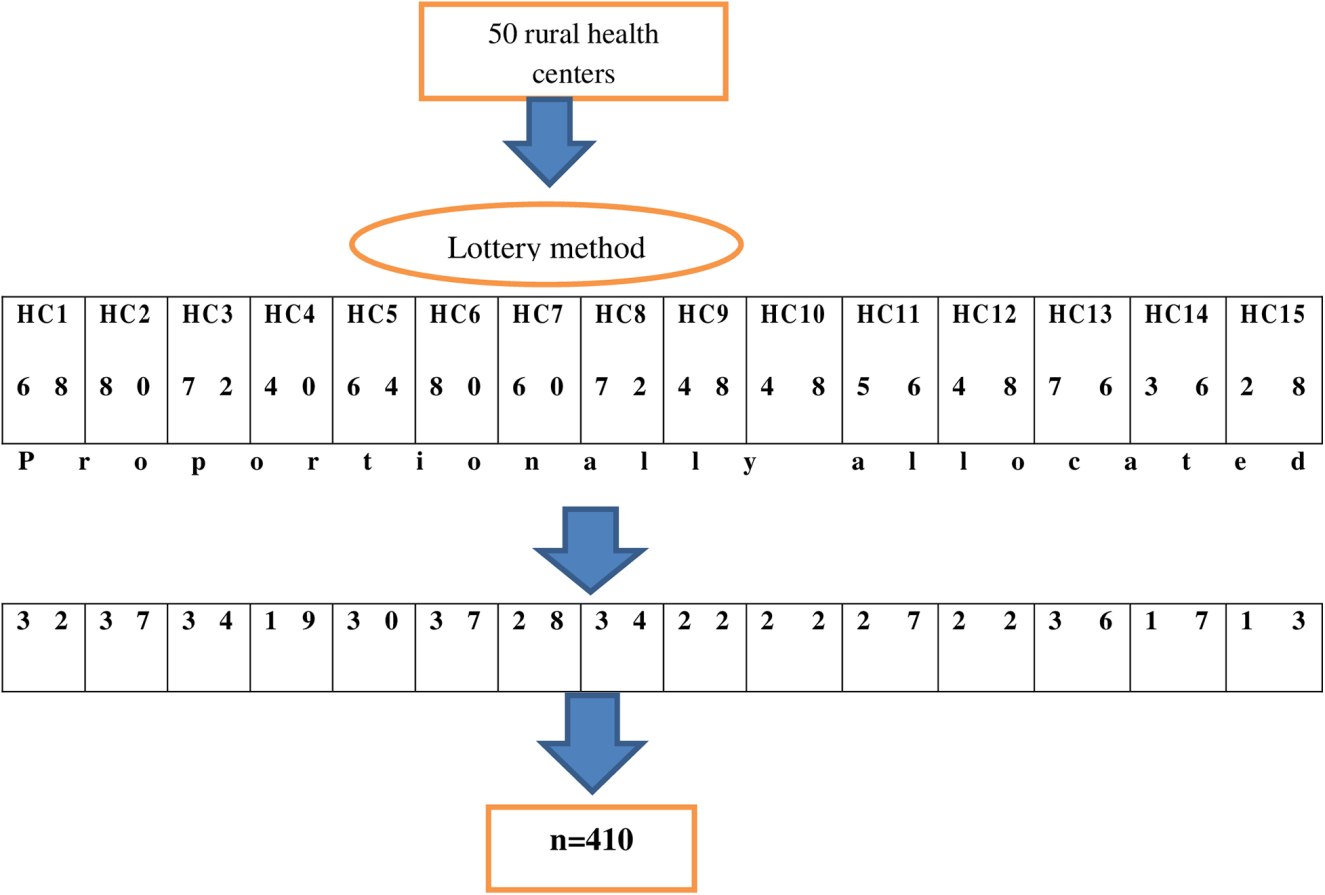
A diagram shows the sampling procedure among mothers who gave birth at rural health centers in Wolaita Zone, Southern Ethiopia.

There were 50 rural health centers in the Wolaita Zone, out of which a subset of 15 health centers were selected for study using the simple random sampling technique. The allocation of participants in the study was done proportionally, taking into account the monthly target delivery performance of the health centers. The study participants were selected from each public health center using the systematic random sampling method (Figure 1).

### Dependent variable

Delay in decision-making to seek delivery at a health facility constituted the dependent variable.

### Independent variables

The independent variables constituted the following:

*Sociodemographic variables*: Mother's age, mother's age at first pregnancy, marital status, mother's educational status, occupational status, place of residence, religion, husband's educational status, and wealth index. *Obstetric factors*: Type of pregnancy (planned/unplanned), gravidity, parity, antenatal care (ANC) service and number of visits, consultation on ANC, history of previous obstetric complications, number of live births, abortion history, number of stillbirths, mode of delivery.

### Operational definition

*First maternal delay in decision-making to seek delivery care*: The time elapsed between the recognition of labor and the decision to seek institutional delivery service. Time spent more than one hour deciding to seek care was considered a delay (22).

*Labor signs and symptoms* are strong and frequent contractions, a bloody show, lower back pain, water breaking, diarrhea or nausea, and a gush or trickle of water as the membranes break contractions (23).

*Knowledge of labor signs and symptoms*: Mothers who can identify two or more signs and symptoms from the list above are considered to have good knowledge (24).

### Data collection tool and procedure

The data collection instrument used in this study was adapted from different pieces of literature previously studied and indicators for maternal health service utilization. To collect the necessary data, a pretested and structured questionnaire was used. The tool was then revised and adjusted based on feedback from supervisors. The principal investigator conducted a detailed orientation session for both the data collectors and the supervisors about an understanding of the data collection instrument and procedure. This session lasted a full day and aimed to provide a comprehensive understanding of the above aspects. The data collection process for this study involved the participation of five BSc midwives and two masters of Public Health as supervisors, with one of the supervisors acting as the principal investigator. Data were collected using face-to-face interview methods.

### Data quality control

The questionnaire was translated from English to the local language (Wolaitegna) and then vice versa. A subset of the sample, consisting of 5% (21 participants), was pretested at a health center not included in the study. Before starting the data collection process, the data collectors were trained on the objectives of the study and the correct method to collect the data using the Open Data Kit. The principal investigator and supervisor conducted daily monitoring. The principal investigator ensured that the data were verified before entry. After daily data collection, the forms were reviewed and assessed for completeness by the principal investigator, who then provided necessary feedback to the data collectors the next morning.

### Data processing and analysis

Data were coded, cleaned, and entered into SPSS for Windows version 25, followed by descriptive and inferential statistical analyses. Descriptive results were presented using tables, charts, and graphs, while a bivariate analysis was used to examine the crude association between each maternal delay and independent variables. Multivariable logistic regression was performed to identify the factors influencing maternal delay in decision-making to seek delivery care at health facilities, entering a variable with a *p*-value < 0.25 in the bivariate analysis. A binary logistic regression analysis was performed to assess the association between independent variables and first maternal delay for seeking delivery care in health facilities, using odds ratios and corresponding 95% confidence intervals (CIs) to express the degree of association. Variables with *P*-values < 0.05 in the multivariable analysis were considered statistically significant.

## Result

### Sociodemographic characteristics of study participants

The study included 398 respondents with a 97.07% response rate. The participants' average age was 26 years old (SD ± 5). Out of the total participants, 256 (64.1%) identified themselves as Protestant Christians and 115 (28.9%) as Orthodox Christians. Furthermore, 276 (69.3%) respondents reported having a formal education. In terms of marital status, 289 (72.6%) of the respondents were either married or living together. In terms of residence, 255 (64.1%) respondents lived in rural areas. The occupational status for most of the mothers included [221 (55.5%)] was housewife. This was followed by 77 (19.3%) farmers and 40 (10.1%) workers. In terms of the occupational status of the men in the family, 199 (50%) of the husbands of mothers were farmers, followed by 86 (21.6%) merchants and 28 (7%) employed. With regard to the wealth index of the study participants, 194 (48.7%) were ranked at the fourth level, indicating a relatively high wealth status. This was followed by 108 (27.1%) at the middle level, 36 (9%) at the second level, and 60 (15.1%) at the poorest level (Table 1).

**Table 1:** Sociodemographic characteristics of study participants at rural health centers in Wolaita Zone, Southern Ethiopia, 2022.

Variables	Categories	Frequency (*n*)	Percentage
Age group	15–20	58	14.6
	21–34	298	74.9
	≥35	42	10.6
Religion	Protestant	255	64.1
	Orthodox	115	28.9
	Muslim	12	3.0
	Others	16	4.0
Mothers’ educational status	Unable to read and write	48	12.1
	Able to read and write	74	18.6
	Primary	204	51.3
	Secondary and above	72	18.1
Marital status	Single	57	14.3
	Married/living together	289	72.6
	Divorced	32	8.0
	Widowed	20	5.0
Mothers’ occupational status	Housewife	221	55.5
	Employee	40	10.1
	Farmer	77	19.3
	Merchant	14	3.5
	Student	21	5.3
	Daily laborer	25	6.3
Husbands’ educational status	Unable to read and write	85	21.4
	Able to read and write	54	13.6
	Primary	129	32.4
	Secondary and above	130	32.7
Husbands’ occupational status	Farmer	199	50.0
	Merchant	86	21.6
	Student	81	20.4
	Daily laborer	4	1.0
	Employee	28	7.0
Residence	Urban	143	35.9
	Rural	255	64.1
Wealth index	Poorest	60	15.1
	Second	36	9.0
	Middle	108	27.1
	Fourth	194	48.7

### Obstetric-related characteristics of study participants

Of the total sample of 398 mothers, 279 (70.1%) were found to have unplanned pregnancies, while 119 (29.9%) had planned pregnancies. A total of 373 mothers (93.7%) attended the ANC follow-up, while 25 mothers (6.3%) did not attend ANC but presented themselves for delivery. Of the mothers who attended ANC, a significant majority of 317 (91%) received ANC counseling. On average, these mothers had three ANC visits, with 348 (93.2%) of them attending less than four times and only 40 (10.8%) attending four or more times. In terms of consultations for institutional delivery, the majority of study participants, 245 (61.6%), sought advice from healthcare professionals, while 153 (38.4%) consulted their families. The majority of deliveries, accounting for 338 (84.9%) cases, were spontaneous vaginal delivery (SVD), while 60 (15.1%) required instrumental assistance. Furthermore, it was observed that 338 (84.9%) mothers had a good knowledge of labor signs and symptoms, while 60 (15.1%) had poor knowledge about them. With regard to the knowledge of obstetric danger signs, 220 (55.3%) mothers had poor knowledge, while 178 (44.7%) showed good knowledge (Table 2).

**Table 2:** Obstetric-related characteristics among mothers who gave birth at rural health centers in Wolaita Zone, Southern Ethiopia.

Variables	Categories	Frequency	Percentage
Planned pregnancy (*N* = 398)	Yes	279	70.1
	No	119	29.9
ANC follow-up (*N* = 398)	Yes	373	93.7
	No	25	6.3
Frequency of ANC visit (*n* = 333)	<4 visit	348	93.2
	≥4 visit	40	10.7
Were they counseled?	Yes	317	91.0
	No	31	8.8
Final decision-maker for institutional delivery service utilization	Husband	124	31.2
	Jointly	140	35.2
	Women	134	33.7
Time spent going to the health facility after detecting first labor pain	≥60 min	164	41.2
	<60 min	234	58.8
Who consulted about institutional delivery service utilization?	Family	153	38.4
	Health worker	245	61.6
Number of children	Primiparous	113	28.4
	Multiparous	285	71.6
Current mode of delivery	SVD	338	84.9
	Instrumental delivery	60	15.1
Heard about obstetric danger signs	Yes	370	93
	No	28	7
Attitude of mothers toward institutional delivery	Negative	189	47.5
	Positive	209	52.5

SVD, spontaneous vaginal delivery.

### Health facility–related characteristics of study participants

Of the 398 study participants, most of them, 162 (40.7%), chose to travel on foot to reach the health center. In contrast, a slightly smaller percentage of participants, 133 (33.4%), chose to use various means of transportation, such as vehicles, to travel to the health center. In addition, a significant proportion of people, that is, 103 (25.9%) of all participants, used the free medical transport service for transport to the health center. Of the participants who arrived at the health facility, the majority (77.9%) of the participants were able to consult an obstetric health provider in the delivery room, while the remaining 22.1% did not. All participants had purchased the necessary items for delivery; however, only 36.4% of them purchased them from a health center, while the remaining 63.6% purchased them from other sources such as private pharmacies and clinics.

A total of 102 mothers had to wait for less than 30 min for availing care. In contrast, the majority (74.4%) of the mothers had to wait for more than 30 min before they could be treated. Concerning their privacy preferences, 297 (74.6%) mothers were screened in the delivery rooms, while a smaller proportion of 15 (3.8%) mothers chose to maintain their privacy by having the door kept closed (Table 3).

**Table 3:** Health facility–related characteristics among mothers who gave birth at rural health centers in Wolaita Zone, Southern Ethiopia.

Variables	Categories	Frequency	Percentage
Means of transportation	On foot	162	40.7
	Ambulance	103	25.9
	Any other means of transport	133	33.4
Payment for transportation	<24.13	305	76.6
	≥24.13	93	23.4
Distance to a health facility in minutes	<60	368	92.5
	≥60	30	7.5
Where did you buy the necessary supplies?	From health center	145	36.4
	Outside of the health center	253	63.6
Served privately/confidentiality of the service	Yes	344	86.4
	No	54	13.6
How much time did you wait?	≥30 min	102	25.6
	<30min	296	74.4

### The magnitude of first maternal delay among mothers at rural health centers in the Wolaita Zone

**Figure 2:**
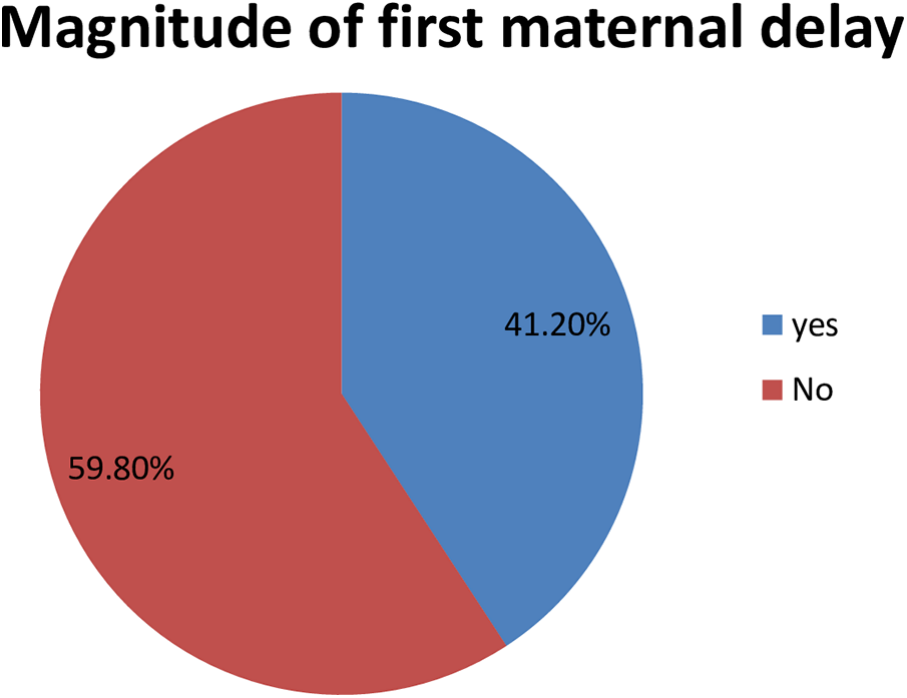
A pie chart shows the magnitude of first maternal delay among mothers who gave birth at rural health centers in Wolaita Zone, Southern Ethiopia.

In this study, the rate of the magnitude of the first maternal delay among mothers who gave birth at rural health centers in the Wolaita Zone was 41.2% (95% CI, 40.09, 44.11) (Figure 2).

### Knowledge about labor signs and symptoms among mothers at rural health centers in the Wolaita Zone

**Figure 3:**
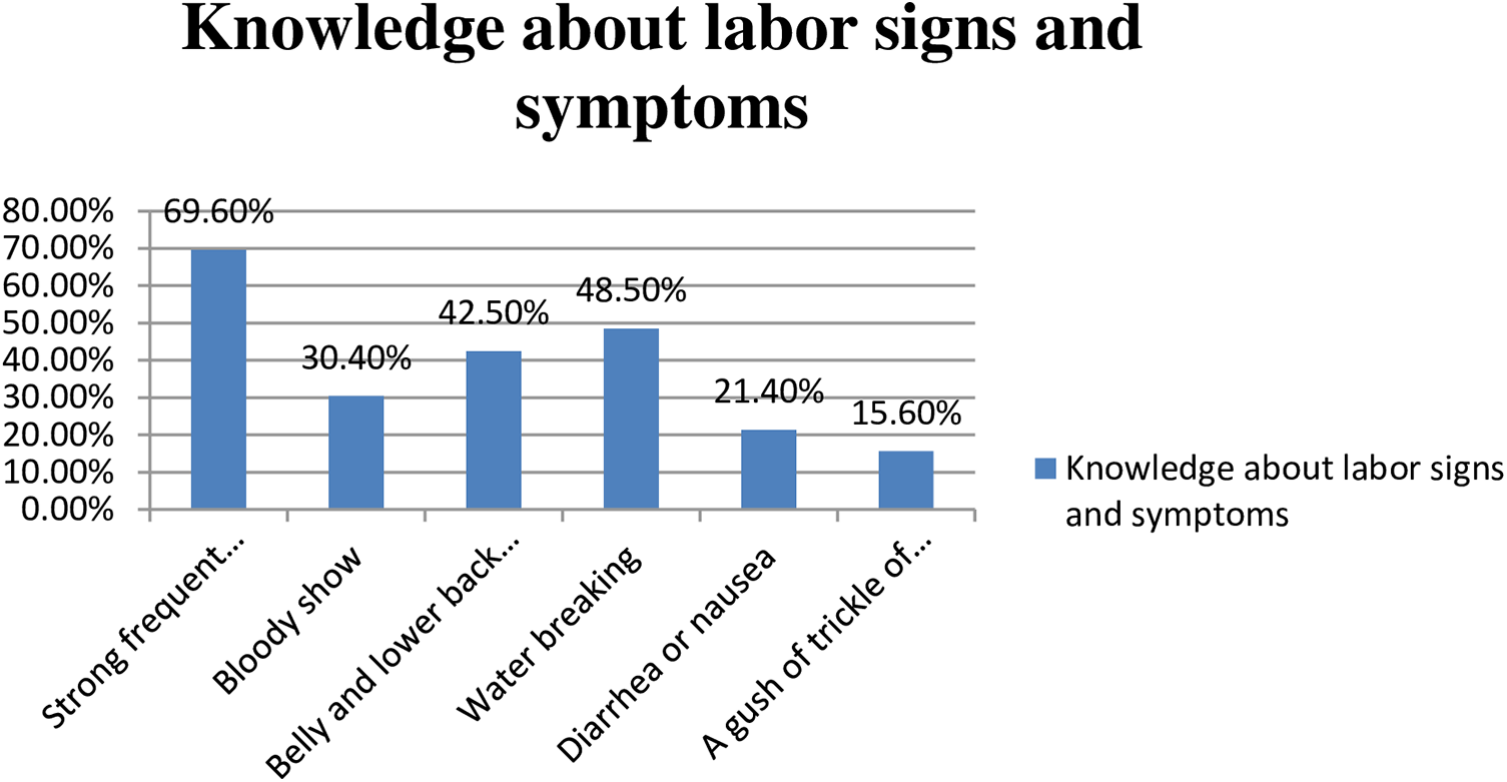
Knowledge about labor signs and symptoms among mothers who gave birth at rural health centers in Wolaita Zone, Southern Ethiopia.

In this study, the commonly reported sign and symptom of labor was the onset of strong and frequent contractions (69.6%), followed by water breaking (48.5%), lower back and abdominal pain (42.5%), bloody show (30.4%), and symptoms of diarrhea or nausea (21.4%). In addition, a gush of fluid was reported in a minority of subjects (15.6%) Figure 3).

### Factors associated with first maternal delay among mothers who gave birth at rural health centers in the Wolaita Zone

In the binary logistic regression analysis, significant associations between various factors and the first maternal delay among mothers were observed. These factors included maternal age, age at first pregnancy, place of residence, occupation, educational status, household income, pregnancy, parity, and planned pregnancy, knowledge of signs and symptoms of labor, knowledge of danger signs, history of complications, and attitude of mothers toward institutional delivery. Factors with a *p*-value <0.25 in the binary logistic regression analysis were considered for a multivariable logistic regression analysis. In the multivariable logistic regression analysis, mothers' first maternal delay in deciding to seek care at health facilities was found to be significantly associated with the following factors: unemployed mothers, husbands with no formal education, and mothers' negative attitudes toward institutional delivery.

In this study, we found that unemployed mothers were 2.5 times more likely to have a delay in deciding to seek care at health facilities than their employed counterparts (AOR, 2.529; 95% CI, 1.546, 4.136). Moreover, it was found that mothers whose husbands had no formal education were almost two times more likely to have a delay in deciding to seek care at health facilities than those whose husbands were educated (AOR, 1.290; 95% CI, 1.029, 1.616).

Finally, mothers who had negative attitudes about seeking care at health facilities were almost two times more likely to have a delay in deciding to seek care at such facilities than those who had good knowledge of seeking care at health facilities (AOR; 1.695; 95% CI, 1.061, 2.709) (Table 4).

**Table 4:** Factors associated with first maternal delay among mothers who gave birth at rural health centers in Wolaita Zone, Southern Ethiopia.

Variables	Categories	First maternal delay		COR (95% CI)	AOR (95% CI)
		Delayed *N* (%)	Not delayed *N* (%)		
Mother's age	15–20	32 (55.2)	26 (44.8)	0.325 (0.139, 0.757)	1.663 (0.850, 3.252)
	21–34	120 (40.3)	178 (59.7)	0.593 (0.292, 1.205)	1.336 (0.718, 2.486)
	>35	12 (28.6)	30 (71.4)	1	1
Mother's age at first pregnancy	<18	81 (37.3)	136 (62.7)	0.703 (0.471, 1.051)	1.457 (0.594, 3.575)
	>18	83 (45.9)	98 (54.1)	1	1
Residence	Urban	51 (35.7)	92 (64.3)	0.697 (0.457, 1.062)	0.651 (0.393, 1.078)
	Rural	113 (44.3)	142 (55.7)	1	1
Mother's education status	No formal education	59 (49.2)	61 (50.8)	1.594 (1.034, 2.455)	1.641 (0.994, 2.707)
	Formal education	105 (37.8)	173 (62.2)	1	1
Husband’s education status	No formal education	70 (50.4)	69 (49.6)	1.781 (1.172, 2.708)	1.290 (1.029, 1.616)*
	Formal education	94 (36.3)	165 (63.9)	1	1
Mother's occupational status	Unemployed	117 (47.8)	128 (52.2)	2.062 (1.347, 3.154)	2.529 (1.546, 4.136)
	Employed	47 (30.7)	106 (69.3)	1	1
Husband's occupational status	Unemployed	115 (38.9)	18 (61.1)	0.687 (0.437, 1.081)	0.619 (0.363, 1.057)
	Employed	49 (48)	53 (51)	1	1
Gravidity	Primigravida	47 (47.5)	52 (52.5)	1.406 (0.890, 2.222)	0.658 (0.158, 2.743)
	Multigravida	117 (39.1)	182 (60.9)	1	1
Parity	Primiparous	57 (50.4)	56 (49.6)	1.693 (1.091, 2.629)	2.859 (0.721, 11.343)
	Multiparous	107 (37.5)	178 (62.5)	1	1
Planned pregnancy	Yes	92 (33)	187 (67)	0.321 (0.206, 0.501)	0.288 (0.168, 0.493)*
	No	72 (60.5)	47 (39.5)	1	1
History of complications	Yes	18 (32.7)	37 (67.3)	0.656 (0.359, 1.199)	0.572 (0.287, 1.142)
	No	146 (42.6)	197 (57.4)	1	1
Attitude of mothers	Negative	92 (48.7)	97 (51.3)	1.805 (1.206, 2.702)	1.695 (1.061, 2.709)*
	Positive	72 (34.4)	137 (65.6)	1	1
Heard about danger signs	Yes	147 (39.7)	223 (60.3)	0.427 (0.194–0.937)	0.630 (0.247, 1.611)
	No	17 (60.7)	11 (39.3)	1	1
Knowledge about labor signs and symptoms	Poor	32 (53.3)	28 (46.7)	1.784 (1.027, 3.098)	1.695 (0.886, 3.242)
	Good	132 (39.1)	206 (60.9)	1	1

* *p*-Value < 0.05.

## Discussion

Improving maternal and newborn health in rural areas of low-income countries is critical to improving global health. Nonetheless, delay in seeking medical care is the leading cause of poor maternal and newborn health and results in preventable deaths worldwide (25). Therefore, this study aimed to assess the magnitude of delay in the decision to seek care at health facilities among mothers who gave birth in rural health centers in Wolaita Zone, Southern Ethiopia.

In this study, it was found that the rate of delay in the decision to seek care at health facilities among mothers who gave birth in rural health centers in the Wolaita Zone of Southern Ethiopia was 42.1%, which was lower than the rates reported in Yem-Special Woreda (26), Myanmar (27), and Brazil (28). This variation could be due to differences in population, research area, timing, cultural factors, healthcare delivery methods, or variations in research methods. The study's finding was in line with previous research conducted in Dawro Zone, Southern Ethiopia, where the prevalence rate was 42% (29). Similarly, a systematic review in Ethiopia reported a consistent prevalence rate of 41% (30), as well as a study conducted in Eritrea, which found a prevalence rate of 42% (31).

In contrast, the present study found a slightly higher prevalence rate compared with previous studies conducted on postpartum mothers in hospitals of the South Gondar Zone in Ethiopia (36.3%) (32), Arsi Zone in Ethiopia (27.2%) (33), and in different regions of Ethiopia (36.1%) (34). The observed disparity could potentially be due to the time gap among the studies conducted. As this study approached the current point, participants’ understanding of obstetric complications improved, along with a corresponding improvement in their attitudes toward using delivery services. Furthermore, the prevalence rate was found to be significantly higher than that of the study conducted in Nepal, which reported a rate of 9.4% (35). This phenomenon could be attributed to differences in sociodemographic and cultural factors.

We found that unemployed mothers had an association with delay in the decision to seek care at health facilities than those who were employed. Unemployed mothers were almost three times more likely to have a delay in the decision to seek care at health facilities than their employed counterparts (AOR, 2.529; 95% CI, 1.546, 4.136). This finding is supported by research done in Jimma Zone, Ethiopia, which revealed that women's limited participation in income-generating activities may contribute to their reliance on their spouse's income, which ultimately influences their decision to seek timely institutional delivery services (36).

Lower or no educational attainment was associated with a delay in the decision to seek care in health facilities and an increased risk of maternal mortality. The husband's educational level was found to be a significant factor contributing to the mother's delay in the decision to seek care in health facilities. It was found that mothers whose husbands had no formal education were almost two times more likely to have a delay in deciding to seek care at health facilities than those whose husbands were educated (AOR, 1.290; 95% CI, 1.029, 1.616). Education has the potential to influence families' perspectives on institutional care and their approach to healthcare utilization. However, individuals with limited literacy skills may experience difficulty accessing and understanding written resources on pregnancy danger signs, birth preparation, complication preparedness, and institutional delivery services (37).

Finally, mothers who had negative attitudes about seeking care at health facilities were almost two times more likely to have a delay in deciding to seek care at such facilities than mothers who had good knowledge of seeking care at health facilities (AOR; 1.695; 95% CI, 1.061, 2.709). It has been observed that health professionals often display negative attitudes toward expectant mothers when seeking care in healthcare facilities. This poor treatment, coupled with the staff's failure to recognize the urgency of mothers' health concerns, was identified as a significant factor influencing their future decision to seek skilled care (38).

### Limitations of the study

This research interview did not include the number of women who died or had significant health complications during childbirth. Consequently, the present analysis may underestimate the magnitude of the delays attributable to the exclusion of these women. In addition, the study's results could be influenced by recall bias regarding timing because mothers were surveyed shortly after birth when they were physically and emotionally exhausted.

## Conclusion

The magnitude of delay in seeking delivery care at health facilities among mothers was high in the study area. Unemployed mothers, husbands with no formal education, and mothers' negative attitudes toward institutional delivery were significantly associated with a delay in the decision to seek care at a health facility. Efforts should be made to strengthen husbands' literacy levels and household income through various income-generating approaches. In addition, the dissemination of health information would raise awareness within the community about the importance of institutional delivery.

## Data Availability

The original contributions presented in the study are included in the article/Supplementary Material; further inquiries can be directed to the corresponding author.
